# Epicardial fat tissue in patients with psoriasis:a systematic review and meta-analysis

**DOI:** 10.1186/s12944-016-0271-y

**Published:** 2016-05-31

**Authors:** Xiaoxue Wang, Zaipei Guo, Zexin Zhu, Yuting Bao, Beichen Yang

**Affiliations:** Department of Dermatovenereology, West China Hospital, Sichuan University, Chengdu, 610041 China; Department of Liver Surgery, Liver Transplantation Division, West China Hospital, Sichuan University, Chengdu, 610041 China

**Keywords:** Epicardial fat tissue, Meta-analysis, Psoriasis

## Abstract

**Background:**

Several studies have been performed to investigate the relationship between psoriasis and epicardial fat tissue (EFT). However, the number of patients of every single study is relatively small.

**Objectives:**

We carried out a meta-analysis to evaluate whether EFT is associated with psoriasis.

**Methods:**

A search of PubMed, Ovid Embase, Ovid Medline, the Cochrane Library and Chinese BioMedical Literature Database (CBM) for controlled trials was done from inception to January 20th, 2016. Published trials that included a psoriasis group and a control group without psoriasis with data for at least epicardial fat tissue (EFT) were included. All statistical analyses were conducted using the Stata 12.0 (Stata Corporation, College Station, TX, USA).

**Results:**

There were 5 trials involving 731 patients. Patients with psoriasis showed significantly higher EFT than control group (SMD: 0.86, 95 % CI: 0.27-1.46, *P* = 0.004).

**Conclusions:**

Patients with psoriasis have higher EFT compared to control subjects without psoriasis.

## Background

Psoriasis is a chronic immune-mediated inflammatory disease which affects 2–4 % of the population worldwide [1]. It is characterized by sharply demarcated erythematous plaque lesions with silvery white scales. A number of literatures pointed that psoriasis is associated with several cardiometabolic co-morbidities and can increase the risk of cardiovascular disease (CVD) and cardiovascular mortality [2–6].

Epicardial fat tissue (EFT) is visceral fat around the heart and coronary vessels. Recent studies suggested that EFT play a significant role in the development of cardiovascular diseases through secreting several inflammatory adipocytokines (TNF-alpha, IL-6, adipocytokines, and leptin) in potential paracrine or endocrine mechanism [7, 8].

Psoriasis and EFT are both associated with CVD. Several studies have been performed to investigate the relationship between psoriasis and EFT [9–13]. But, the number of patients of every single study is relatively small. In this study, we play a meta-analysis to evaluate whether EFT is associated with psoriasis.

## Materials and methods

The process of the meta-analysis was performed according to the Cochrane Collaboration recommendations [14]. The analytical results were reported according to the PRISMA (Preferred Reporting Items for Systematic Reviews and Meta-Analyses) statement [15].

### Search strategy

A systematic search was made in the following databases: PubMed, Ovid Embase, Ovid Medline, the Cochrane Library and Chinese BioMedical Literature Database (CBM). The search used the terms “epicardial fat tissue”, “psoriasis [Mesh]” which were searched as text words and as exploded medical subject headings where possible. Free text words were searched combined with additional keywords: “epicardial adipose tissue”, “epicardial fat”, “epicardial adipose”. No language restrictions were applied. The search included literatures published until January 20th, 2016 with no lower date limit.

### Data extraction and quality assessment

Data were abstracted and quality of studies was assessed independently by two reviewers (Xiaoxue Wang, Zexin Zhu). Disagreement on specific studies between the two reviewers was resolved by consensus. Data were extracted from each study including the sample size and mean difference ± SD in EFT, total cholesterol, low-density lipoprotein (LDL), high-density lipoprotein (HDL), triglycerides in both psoriasis and non-psoriasis groups. Methodological study quality was assessed using the Strengthening of Reporting of Observational Studies in Epidemiology (STROBE) checklist of 22 items [16].

### Including and excluding criteria

Studies investigating the association between EFT and psoriasis were considered for this meta-analysis. And the diagnosis of psoriasis was made according to clinical or pathological examination. The studies should include at least a psoriasis group and a control group. Abstracts, letters, and case reports and studies without sufficient formation on EFT were excluded. If the same samples were used in multiple publications, the most recent and/or the largest publication were included.

### Statistical analyses

All statistical analyses were carried out using Stata 12.0 (Stata Corporation, College Station, TX, USA). Pooled standard mean difference (SMD) or weighted mean difference (WMD) with 95%CI were calculated using either the fixed-effects model or random-effects model. For each meta-analysis, the *χ*^2^ and I^2^ statistics were first calculated to assess the heterogeneity of the included studies. *P* < 0.1 and I^2^ > 50 % were considered significant. For *P* < 0.1 and I^2^ > 50 %, the random-effects model was utilized. Otherwise, data were evaluated using the fixed-effects model. The risk of publication bias in this study was evaluated by visual inspection of the symmetry of the funnel plot. The significance of the pooled SMD or WMD was rated by the Z-test. *P* < 0.05 was regarded as significant.

## Results

### Description of the studies

**Fig. 1:**
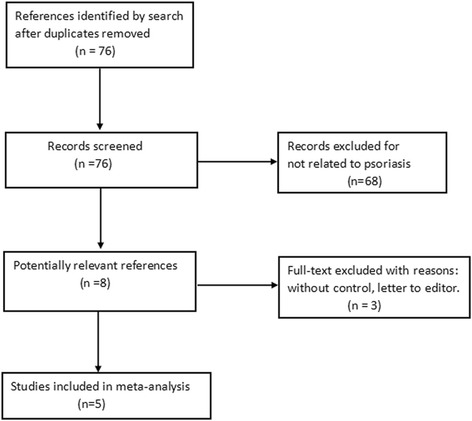
Flowchart showing selection of publications

We have searched a total of 76 studies, and 5 studies left after excluded. The full-texts had been carefully evaluated. They were published from 2013 to 2015, all of them measured EFT in psoriasis and control group [9–13]. Totally 731 patients were enrolled in these studies. Among those, 349 patients with psoriasis compared with 382 subjects without psoriasis (Fig. 1).

The patients in each study are ranging from 63 to 302. EFT thickness was measured by echocardiography in 3 studies [9–11]. EFT was calculated as the area [12] or volume [13] by computed tomography (CT) in 2 studies. Disease severity of psoriasis was quantified using the Psoriasis Area and Severity Index (PASI) in all included studies. These characteristics and quality assessment assessed by the STROBE are listed in the Table 1.

**Table 1:** The characteristics of studies included in the meta-analysis

Study	Arms	Patients	Gender (M/F)	Age (year)	BMI (Kg/m^2^)	PASI score	Total cholesterol, mg/dl	Triglycerides, mg/dl	HDL, mg/dl	LDL, mg/dl	EFT	Measurement tool	Study quality
Sen. et al. 2013 [9]	Psoriasis	65	39/26	41.1 ± 3.3	25.9 ± 2.8	17.5 ± 5.7	190 ± 46	163 ± 78	33 ± 9	132 ± 33	7.3 ± 0.5 mm	Echocardiography	20
	Non-psoriasis	50	28/22	40.5 ± 3.8	25.5 ± 2.3		199 ± 37	172 ± 88	34 ± 7	121 ± 36	6.5 ± 0.5 mm	Echocardiography	
Zehra. et al. 2014 [10]	Psoriasis	31	14/17	42 ± 11.1	28.1 ± 5.6	6.1 ± 4	206.1 ± 34.8	165.4 ± 75.3	47.9 ± 8.0	125.1 ± 30.5	6.4 ± 2.6 mm	Echocardiography	19
	Non-psoriasis	32	13/19	41.1 ± 6.8	30.9 ± 9.9		198.6 ± 31.8	143.5 ± 60.6	43.3 ± 13.4	126.6 ± 28.0	5.1 ± 1.9 mm	Echocardiography	
Ahmet. et al. 2014 [11]	Psoriasis	115	62/53	33.6 ± 6.0	26.1 ± 3.1	3.8 ± 4.1	185.4 ± 37.4	125.3 ± 77.3	49.2 ± 12.9	129.4 ± 33.7	5.7 ± 1.2 mm	Echocardiography	20
	Non-psoriasis	60	28/32	32.5 ± 8.3	25.2 ± 3.2		179.1 ± 33.8	126.2 ± 67.0	48.4 ± 13.5	127.7 ± 26.1	4.1 ± 1.0 mm	Echocardiography	
Balci .et al. 2014 [12]	Psoriasis	38	26/12	42.2 ± 15.0	28.8 ± 3.9	9.8 ± 9.3	203.1 ± 33.7	150.2 ± 74.4	136.2 ± 27.7	37.6 ± 9.9	13.8 ± 8.4 cm^2^	CT	19
	Non-psoriasis	38	26/12	39.8 ± 13.5	27.4 ± 4.2		185.2 ± 38.5	130.1 ± 62.6	124.4 ± 42.1	41.4 ± 8.9	9.7 ± 6.4 cm^2^	CT	
Torres. et al. 2015 [13]	Psoriasis	100	64/36	47.4 ± 10.8	28.6 ± 4.99	12.8 ± 8.0	206.5 ± 39.9	126.7 ± 72.6	129.1 ± 38.2	51.5 ± 13.2	101.4 ± 55.52 ml	CT	21
	Non-psoriasis	202	130/72	54.4 ± 10.1	28.1 ± 4.17		NA	NA	NA	NA	92.2 ± 38.33 ml	CT	

Continuous variables were presented as mean ± standard deviation (SD), and categorical variables were presented as frequencies with percentages

*BMI* body mass index, *PASI* psoriasis area and severity index, *LDL* low- density lipoprotein, *HDL* high-density lipoprotein, *CT* computed tomography, *NA* not applicable

We compared the EFT, serum level of total cholesterol, LDL, HDL and triglycerides.

### Epicardial fat tissue

**Fig. 2:**
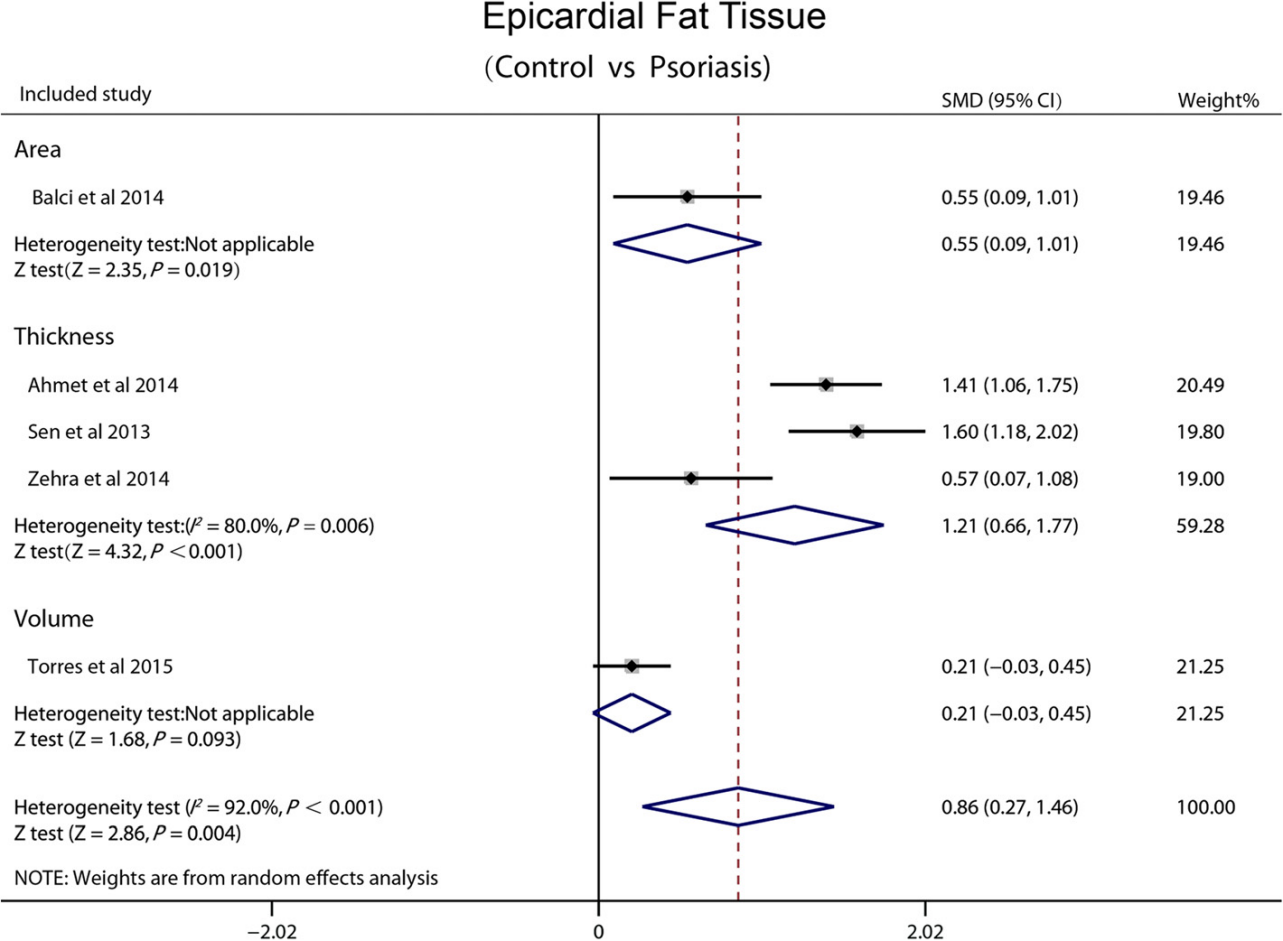
Forest plot showing standardized difference in means and 95 % CI of EFT between patients with psoriasis and control group

All studies included reported EFT. Subgroup analysis was performed depending on different measurement of EFT (thickness, area and volume). Based on the great heterogeneity between trials (I^2^ = 92 %, *P* < 0.001), random effects meta-regression analysis was made. There was a significant difference in the SMD between psoriasis and non-psoriasis groups (0.86, 95 % CI: 0.27-1.46, *P* = 0.004) (Fig. 2).

### Total cholesterol

**Fig. 3:**
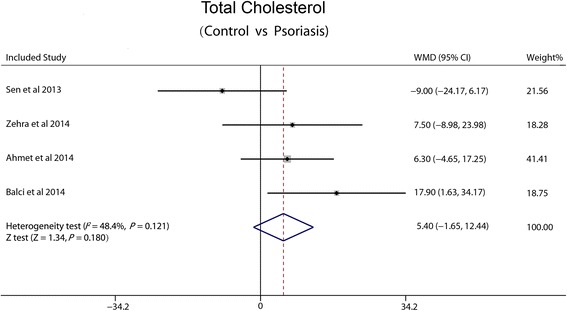
Forest plot showing difference in means and 95 % CI of total cholesterol between patients with psoriasis and control group

Four studies reported the total cholesterol in the serum. We made a fixed-effect pooled analysis based on the low heterogeneity among the 4 trials (I^2^ = 48.4 %, *P* = 0.121). There was no significant difference in the WMD between psoriasis and non-psoriasis groups (5.40 mg/dl, 95 % CI: −1.65-12.44, *P* = 0.18) (Fig. 3).

### Low-density lipoprotein

**Fig. 4:**
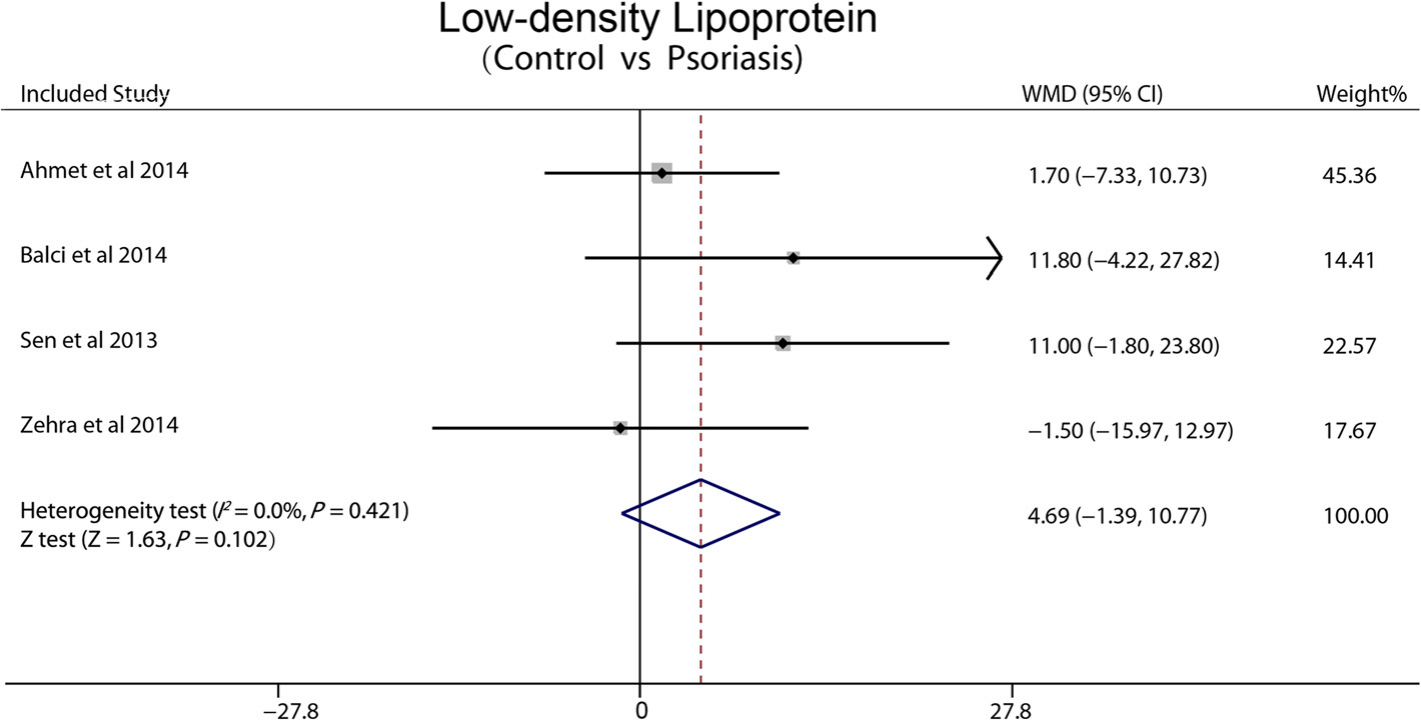
Forest plot showing difference in means and 95 % CI of LDL between patients with psoriasis and control group

Four studies included reported LDL. According to the low heterogeneity between trials (I^2^ = 0 %, *P* =0.421), fixed-effect pooled analysis was made. There was no significant difference in the WMD between psoriasis and non-psoriasis groups (4.69 mg/dl, 95 % CI: −1.39-10.77, *P* = 0.102) (Fig. 4).

### High-density lipoprotein

**Fig. 5:**
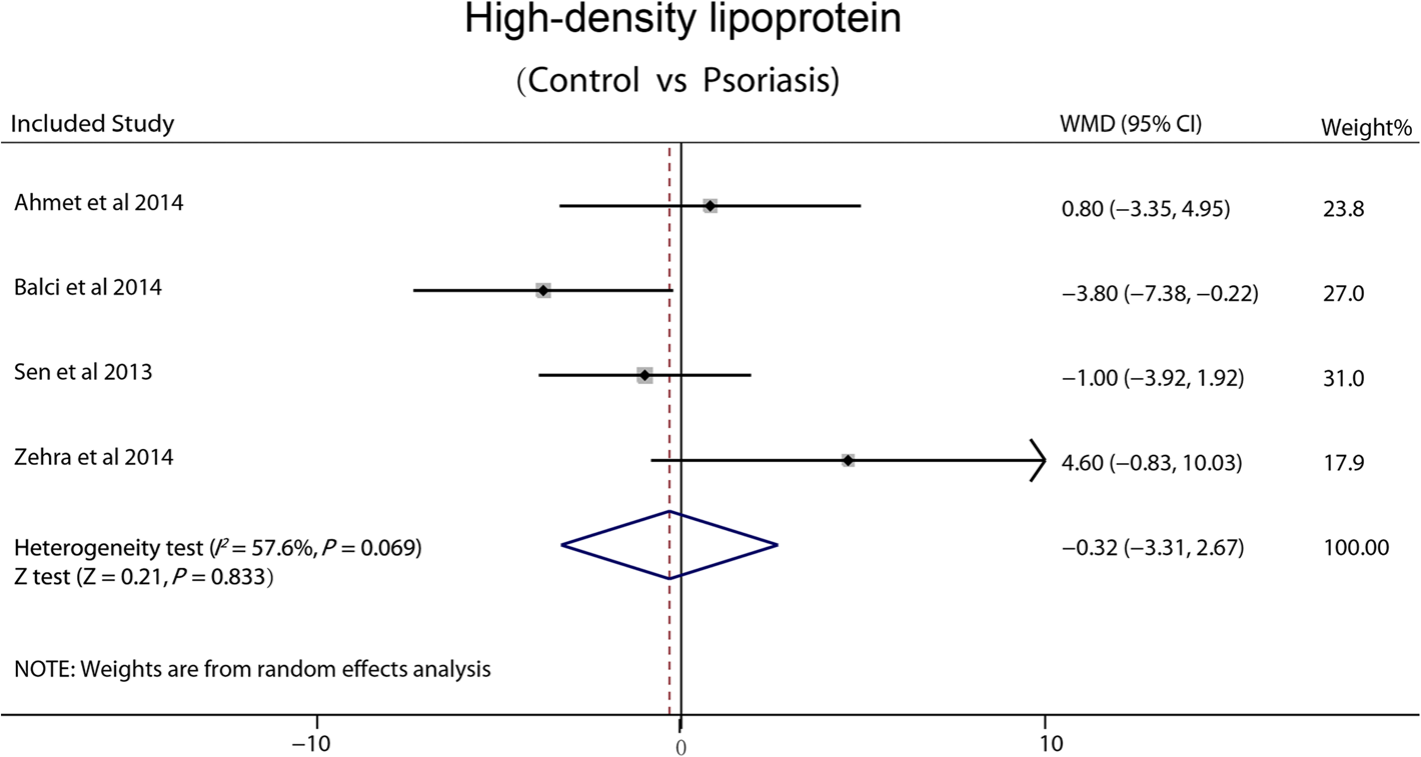
Forest plot showing difference in means and 95 % CI of HDL between patients with psoriasis and control group

Four studies reported HDL. Heterogeneity between trials (I^2^ = 57.6 %, P = 0.069) is high, so random effects meta-regression analysis was made. There was no significant difference in the WMD between psoriasis and non-psoriasis groups (−0.32 mg/dl, 95 % CI: −3.31-2.67, *P* = 0.833) (Fig. 5).

### Triglycerides

**Fig. 6:**
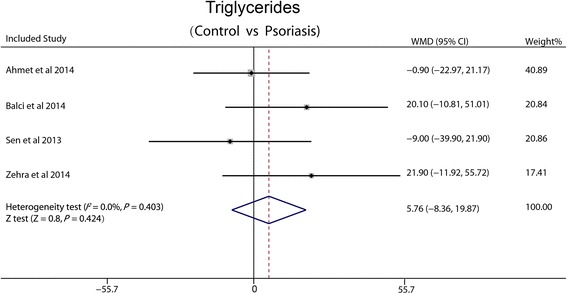
Forest plot showing difference in means and 95 % CI of triglycerides between patients with psoriasis and control group

Four studies included reported triglycerides. Based on the low heterogeneity between trials (I^2^ = 0 %, *P* = 0.403), fixed-effect pooled analysis was made. There was no significant difference in the WMD between psoriasis and non-psoriasis groups (5.76 mg/dl, 95 % CI: −8.36-19.87, *P* = 0.42) (Fig. 6).

Publication bias was not assessed because of limited studies.

## Discussions

The results of this meta-analysis suggest that EFT increased significantly in psoriasis patients, compared with non-psoriasis patients. However, there was no significant difference in total cholesterol, LDL, HDL and triglycerides between the patients with and without psoriasis.

In recent years, many clinical studies found that psoriasis patients are under a high risk of CVD. Neimann et al. [17] conducted an epidemiology investigation of the relationship between psoriasis and coronary heart disease based on the General Practice Research Database (131,560 psoriasis patients and 465,252 non-psoriasis patients). This study showed that traditional risk factors for coronary heart disease such as diabetes, hypertension, hyperlipidemia, obesity and smoking are highly correlated with psoriasis. A prospective study showed that patients with psoriasis is under a higher relative risk (RR) of CVD compared with the general population [18]. Alexandroff et al. put forward that psoriasis is an independent risk factor for myocardial infarction (MI) [19]. CVD and psoriasis have similar histology and molecular immune pathological basis [20], based on Th1 cytokines (TNF alpha, IL - 2, IL - 10, IL - 12) [21].

Epicardial fat tissue (EFT) refers to the visceral fat deposition in heart, and it developed from brown adipose tissue in embryonic period. EFT has both protective effects and adverse effects [22]. Its physiological role has not been completely elucidated so far. Clinical research has proved that the EFT is associated with cardiovascular risk factors. Epicardial fat thickness in the metabolic syndrome population is significantly higher than that of control group [23, 24]. EFT is the source of cytokines which can induce mononuclear cells infiltration into the vascular intima. This process is an important step in the development of atherosclerosis. The expression of inflammatory gene in EFT is higher than that in the subcutaneous adipose tissue in cardiovascular disease patients. And EFT release more inflammatory mediators such as, interleukin, MCP - 1 and TNF alpha [25]. The thickness, area or volume of EFT can be measured and evaluated by two-dimensional echocardiography, CT or magnetic resonance imaging (MRI).

To our knowledge, there are few studies explored the relationship between EFT and psoriasis. However, the causes of increased EFT in psoriasis patients are still unknown. Chronic immune-mediated systemic inflammation may be liable for this phenomenon. This meta-analysis also showed that no significant difference was existed in the serum level of total cholesterol, LDL, HDL and triglycerides between psoriasis patients and control subjects. This demonstrates that increased EFT may be an independent risk factor for psoriasis.

There is no multi-center, large sample size study about the role of EFT in psoriasis published, and this meta-analysis is the first time to investigate the relationship between EFT and patients with psoriasis. This study may have several possible limitations. Firstly, three studies included [9, 10, 12] were small sample size. Secondly, all the studies were performed as the cross sectional design and the study groups were not under clinical follow-up. Thirdly, the gold standard in measuring EFT is MRI. Echocardiography is less accurate than CT or MRI, but echocardiography is a noninvasive, less costly and convenient method. Another possible limitation of this analysis is the great heterogeneity of EFT between trials. That may be caused by differences in measurement technology.

## Conclusion

In conclusion, this meta-analysis has demonstrated that there is no significant difference in total cholesterol, LDL, HDL or triglycerides between psoriasis and control, but higher EFT in psoriasis group compared to control group. That may indicate that EFT is an independent risk factor of psoriasis. This might give an explanation for the relationship between psoriasis and elevated risk of CVD. Moreover, increased EFT can also provide a new clue for the future research of psoriasis.
